# Risk assessment and evaluation of heavy metals concentrations in blood samples of plastic industry workers in Dhaka, Bangladesh

**DOI:** 10.1016/j.toxrep.2020.10.003

**Published:** 2020-10-09

**Authors:** Md. Shakil Ahmed, Mahbuba Yesmin, Farah Jeba, Md Sirajul Hoque, Ahsan Rahman Jamee, Abdus Salam

**Affiliations:** a Department of Chemistry, Faculty of Science, University of Dhaka, Dhaka 1000, Bangladesh; b Department of Medicine, Enam Medical College & Hospital, Savar Dhaka, Bangladesh; c Department of Soil, Water and Environment, Faculty of Biological Sciences, University of Dhaka, Dhaka 1000, Bangladesh; d Department of Statistics, Faculty of Science, University of Dhaka, Dhaka 1000, Bangladesh

**Keywords:** Heavy metals, Human blood, Industrial dust, Hazard index, Carcinogenic risk

## Abstract

To assess the potential health risk caused by heavy metals twenty-six blood samples were collected from plastic industry workers based on ages and smoking status in Dhaka, Bangladesh. Heavy metals were analyzed with an atomic absorption spectrometer. The mean concentrations of Lead (Pb), Cadmium (Cd), Nickel (Ni), and Zinc (Zn) found in blood samples of the exposed workers were 32.78 ± 9.47, 1.08 ± 0.47, 1.42 ± 1.01, and 9.08 ± 1.95 μgL^−1^, respectively. The average heavy metal concentrations in blood samples of smoking workers show a narrow range of fluctuation than that of non-smoking workers. A review of different age groups of industry workers shows the workers between the ages of 26 and 40 are more likely to contaminated with Pb (35.90 ± 8.06 μgL^−1^) and Ni (1.61 ± 1.31 μgL^−1^). The higher level of Cd (1.26 ± 0.46 μgL^−1^) and Zn (9.91 ± 2.80 μgL^−1^) was found in >40 years old workers. The mean concentration in indoor dust samples of different industrial subsections reported as 40.27 ± 10.33, 3.24 ± 0.83, 18.08 ± 3.61, and 103.64 ± 8.16 mg kg^−1^ for Pb, Cd, Ni, and Zn, respectively. Exposed workers have relatively less critical health implications concluded from the average daily intake (ADI), hazard quotient (HQs), and hazard index (HI) values. The HI values of Pb, Cd, Ni, and Zn were reported as 2.0 × 10^-2^, 4.64 × 10^-4^, 1.62 × 10^-3^, and 5.49 × 10^-4^, respectively, which have imparted minimal risks (as HI < 1) to the health of the workers. The cancer risks of Pb, Cd, and Ni were reported as 1.46 × 10^-10^, 1.77 × 10^-9^, and 1.31 × 10^-9^, respectively lower than the threshold values. Therefore, the result divulged a potentially lower cancer risk compared to EPA limit value of 1 × 10^-6^ to 1 × 10^-4^ for exposed industrial workers.

## Graphical abstract

**Figure:**
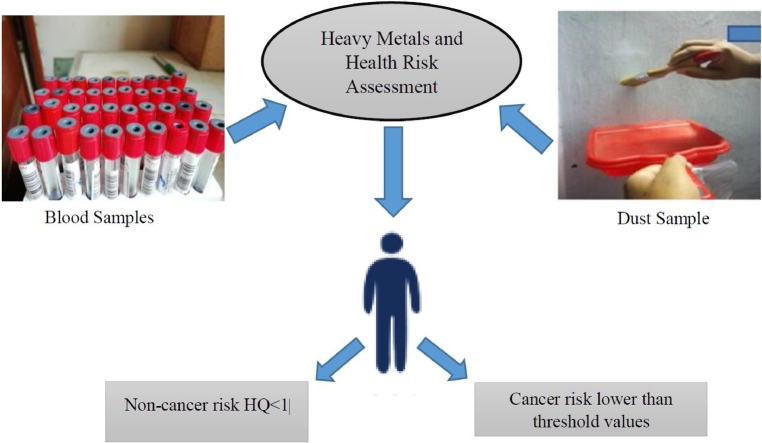

## 1 Introduction

Plastic materials are widely used in industry and have become an integral part of our daily life and its use is increasing day by day. It has been growing at about 15 percent a year on the back of spiraling demand from domestic and export markets where annual sales of plastic products in Bangladesh are currently estimated at around Tk 150 billion in the local market. Several types of extrusion materials, molding, thermosetting conversion like manufacturing of PVC, shopping bag, PET/PE bottles, laminating packages, rigid sheets, plastic shoes, household products, etc. are produced in various plastic industries. Plastic materials contain a wide variety of additives (plasticizers, antioxidants, stabilizers, curing agents, coloring agents, etc.) to fulfill their physical and chemical properties [1,2]. Metals such as lead (Pb), mercury (Hg), chromium (Cr), cadmium (Cd), nickel (Ni), and zinc (Zn) are found in plastic composites, as fillers and colorants [3]. Zinc oxide (ZnO) [4], zinc sulfide (ZnS), lead carbonate (PbCO_3_) typically used in 0.01–10 % (w/w) as inorganic pigments and fillers in plastic materials [3]. Heat stabilizers mainly use PVC, based on Pb, Cd, Sn, and Zn compounds and for flame retardants, zinc borate (Zn_3_B_2_O_6_) is used [3] in plastic materials. Metals in plastic materials are loosely bound to the surface and can be easily leached to the environment as plastic wastes and particulate matter dust [1]. The dust emitted from the processing of the plastic materials contains heavy metals and an exorbitant of epidemiological and toxicological evidence from all around the globe has shown a variety of health risks to human populations related to environmental, occupational and dietary exposure to such metals, causes pulmonary and systemic inflammation which shows a plausible relationship between air pollution and blood clotting in the circulatory system. [[5], [6], [7], [8]]. Air pollutants, inhaled or absorbed by dermal contacts in an industrial environment, usually contain several chemicals contaminated with heavy metals. Plastic wastes are being recycled in many developing countries especially in Asia, Africa and Latin America through some of the primitive methods and technologies which are not environmentally friendly, safe, and suitable [9]. Occupational exposers to these heavy metals cause extensive health deteriorations, such as asthma, back pains, bronchitis, chronic dermatitis, hypertension, kidney diseases, and even cancer [[10], [11], [12]]. Heavy metals such as Pb, Cd, and Ni have been known as mutagenic to human health [13,14]. Chronic exposure to toxic metals can increase the body's production of reactive oxygen species (ROS), leading to oxidative stress induction, induce mitochondrial DNA mutations, reduces the mitochondrial respiratory chain functions, the permeability of cellular membrane being altered which causes the deterioration of mitochondrial antioxidant system and causing significant damage to cellular components such as lipids, proteins, and deoxyribonucleic acid (DNA) [[15], [16], [17]].

Regular monitoring and assessment of heavy metals have been carried out in almost all the developed and developing countries [1], but a limited number of reports have been published on the assessment of heavy metals in the plastic industry workers bio-matrices. A further, carcinogenic risk has been predicted to be an increasingly important cause of mortality in Bangladesh in the next few decades [18]. In bio-monitoring studies, the blood samples are widely used as conventional bio-marker for heavy metal analysis [19,20]. Assessments of toxic heavy metals content have performed in smoker blood samples of three different age groups (≤25, 26–40, and >40 years) in Taif city, Saudi Arabia. The average concentrations of Cd, Pb, Hg, Mn, Zn, Cr, and Ni in blood samples of these three groups were 1.8, 23.2, 2.8, 6.5, 4288, 179, and 164.5, μgL^−1^, respectively [21]. Generally, the assessment is carried out based on different sets of categories (occupation, sex, food, drink and smoking habits, age groups etc.) [22]. However, the information regarding the heavy metals level in the blood of the plastic industry workers and associated health effects are still very poor in Bangladesh.

Therefore, this study focuses on the assessment of toxic heavy metals in blood specimen and indoor plastic industrial dust, and evaluate the impacts of industrial environmental contamination on the health status of workers based on carcinogenic (Cd and Pb) and non-carcinogenic (Pb, Cd, Ni, and Zn) risk factors. It is worth mentioning that the International Agency for Research on Cancer (IARC), cadmium (Cd) is labeled as a group 1 carcinogenic to humans, and lead (Pb) is a group 2A plausible human carcinogen. [23].

## 2 Materials and methods

### 2.1 Sampling location and description of the industry

**Fig. 1:**
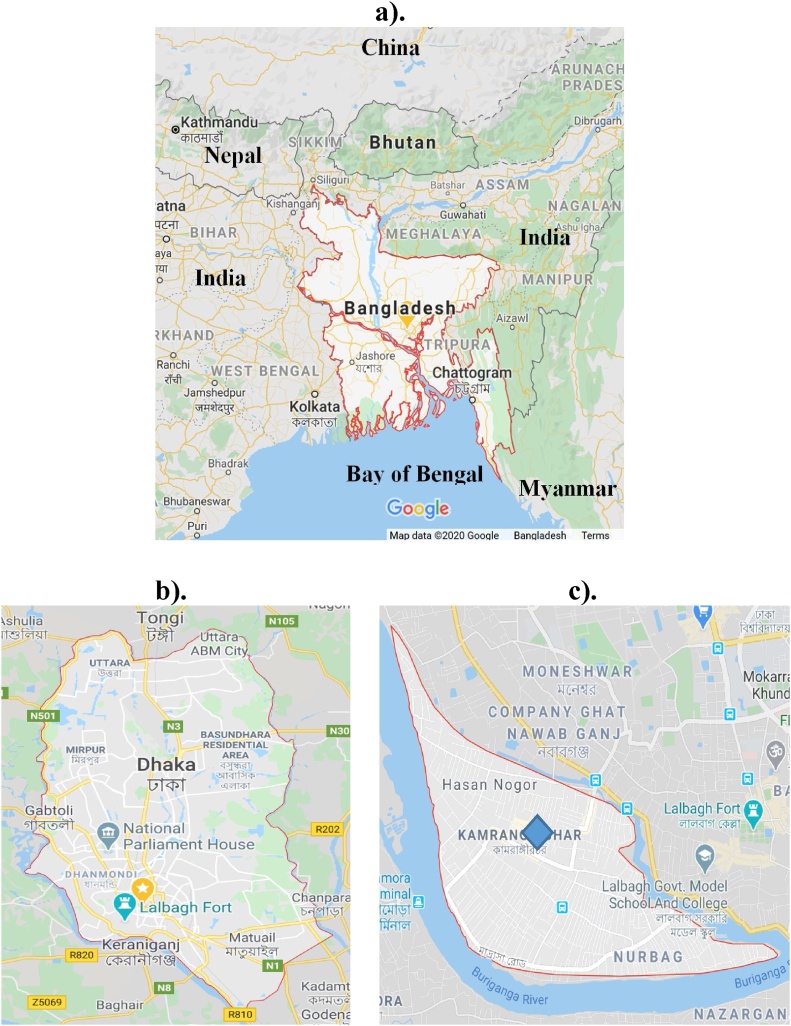
a) Map of Bangladesh (Source: Google); b). Map of Dhaka Mega City, c). Map of the Plastic Industry location in Kamrangirchar [] Dhaka, Bangladesh.

Bangladesh (Fig. 1) is one of the densely populated and the most polluted countries in the world – every year thousands of people were dying due to environmental pollution ([41,44]). Dhaka is the capital city with all the problems of an unplanned megacity. It has long been regarded as a commercial as well as an industrial city. The blood samples were collected from a plastic industry workers at Kamrangirchar (a crowded area in the southern part of Dhaka), which is located between latitude 23.71822° and longitude 90.36777°. Plenty of small and large plastic factories have sprung up here to compete with other small scale industries. A major Buriganga river flows by the close proximity of the sampling area, which is highly contaminated with industrial wastes and effluents. The selected plastic industry for the current study is situated at the center of the whole industrial zone.

It is the largest one among all the plastic industries in this region. This industry produced plastic bags, bottles, large containers, and other plastic materials depending upon the demand of the market. This industry also has a recycling unit for the plastic materials. However, sixty workers along with six management personnel including both men and women are involved in the daily operation of the industry. The main sections of the industry were raw materials unit, plastic recycling unit, and quality control and production unit with an office space of the management personnel.

### 2.2 Sample collection and storage

To determine the contamination level of heavy metals healthy workers were recruited voluntarily for blood samples with personal and medical histories, along with relevant details of the subjects that were taken for the study through a questionnaire survey. Samples were collected in an evacuated (vacutainer) blood-collection tube each of which contains 4 mL of blood. Before collecting blood, the skin of the arm of the person was cleaned with double distilled water and analytical grade ethanol (the latter without cotton, just a rinse), and allow to dry by evaporation. The blood sample was stored, allowed to clots and serum was separated by centrifugation. The serum was discharged into a new clean tube. Whole blood was used for determining Cd and Pb, serum was used for other metals [[24], [25], [26]]. Toxic trace elements as Pb and Cd bond preferably on erythrocytes, therefore for the determination of Pb and Cd, the whole blood was preferred [27].

### 2.3 Heavy metals extraction and determination

Conventional Wet Acid Method was used for blood sample digestion [28]. Accurately 0.5 mL of serum (for Ni and Zn) and 1.0 mL of whole blood (for Pb and Cd) were taken into small beaker separately. 10.0 mL of freshly prepared concentrated (65 %) nitric acid was added into each beaker and stood for 10 min. The beakers were covered with a watch glass and then digested at 60–70 °C for one and a half hours. The digests were then treated with 2 mL nitric acid, while heating continued on a hot plate at about 80 °C until a clear digested solution was obtained. The excess acid was evaporated to semi-dry mass, cooled and diluted with 0.1 mL nitric acid. These were transferred into a 50.0 mL volumetric flask and diluted to mark using distilled water. The concentrations of heavy metals (Zn, Cd, Ni, and Pb) were determined with an Atomic Absorption Spectrophotometer (AAS). Standard solutions of each metal (Zn, Cd, Ni, and Pb) were prepared according to the procedure for AAS to be used for the calibration curve [29]. Attempts were made to determine chromium (Cr) concentrations in all the samples, but Cr level was below the detection limit in each case. The limit of detection (LoD) and limit of quantification (LoQ) of these metals have been calculated (Table 1) according to Shrivastava [30] with the formulas of LoD = 3.3 S_a_/b, and LoQ = 10 S_a_/b; where, Sa is the standard deviation of y-intercept (absorbance) of linear regression lines and b is the slope of the calibration curve (absorbance vs concentration).

**Table 1:** The limit of detection (LoD) and limit of quantification (LoQ) values of each metals.

Elements	LOD	LOQ
Pb	0.277	1.330
Cd	0.110	0.496
Ni	0.026	0.077
Zn	0.040	0.147

### 2.4 Quality control and limitation of the study

Quality control and quality assurance procedure has been maintained throughout the sampling and also during metal analysis in the Atomic Absorption Spectrophotometer (AAS). The intensity of the hollow cathode lamp, temperature and humidity of the instrument room was maintained within limits of AAS operating protocol. The calibration curve was constructed for each metal with five standard solutions of known concentration by covering the linear range. The concentration of each metal was measured three times and took the average. Blank samples (field and reagent blanks for both blood and dust samples) were collected and treated as the same procedures of the real samples. Blank corrections were done by deducting the blank value of the metal from the real sample concentration for each metal. The current study was limited to five heavy metals and in one plastic industry. We are planning to conduct another study in the future with more toxic metals from many industries with an increased number of sample sizes.

### 2.5 Dust sample collection and analysis

Indoor industrial dust samples were also collected for risk assessment. The samples were procured randomly from different sections of the industry (i.e. plastic recycling unit - P1, pellet manufacturing – P2, and production unit – P3) using a small plastic brash and a small plastic scoop, and stored in labeled plastic envelopes. The samples were stored at room temperature in the laboratory, air-dried for 48 h, and ground with mortar and pestle. The samples were then passed through a conventional sieve to remove the coarse particles larger than 90 μm. A mass of 1.0 g of oven-dried dust samples was kept in acid digestion, cooled, filtered with Whatman filter paper and analyze with AAS. Calibration of the instrument was also performed using a set of standards for dust samples. Using the calibration curve, the concentrations of heavy metal were determined in the unit of mgkg^−1^.

### 2.6 Non-carcinogenic risk estimation

Non-carcinogenic risk is defined as something that is not known to cause cancer. It is characterized by a term called hazard quotient (HQ), which is the probability of an individual suffering from an adverse effect [46]. The term HQ is expressed as the quotient of acceptable daily intake (ADI) divided by the chronic reference dose (RfD) of a specific heavy metal [46].

(1) $$\text{H}\text{Q}\;=\frac{ADI}{RfD}$$

Where ADI is expressed by three different pathways (ingestion, inhalation, and dermal contact) [31] through which a specific toxic heavy metal intakes in the human body [47].

(2) $$D_{\text{i}\text{n}\text{g}\text{e}\text{s}\text{t}\text{i}\text{o}\text{n}}=\frac{C\times IngR\times EF\times ED\times CF}{BW\times AT}$$

(3) $$D_{\text{i}\text{n}\text{h}\text{a}\text{l}\text{a}\text{t}\text{i}\text{o}\text{n}}=\frac{C\times inhR\times EF\times ED}{PEF\times BW\times AT}$$

(4) $$D_{\text{d}\text{e}\text{r}\text{m}\text{a}\text{l}}=\frac{C\times SA\times AF\times ABS\times EF\times ED\times CF}{BW\times AT}$$

where, C is the chemical concentration of metal in the medium *i.e*. dust (mg kg^−1^); IngR is the rate of ingestion (mg day^−1^); InhR is the inhalation rate (m^3^ day^−1^); SA, Skin area exposed to heavy metal (cm^2^); AF is the skin adherence factor (mg cm^-2^ day^−1^); EF represent the frequency of exposure (day year^−1^); ED stands for the duration of exposure (years) to a specific medium; PEF is the particle emission factor (m^3^ kg^−1^); SL is the skin adherence factor (mg cm^-2^ day^−1^); ABS is the dermal absorption factor; BW is the average body weight (kg) of the worker; AT is the average time period. The reference values were given in Table 2 for the above parameters.

**Table 2:** Recommended values of the parameters used to calculate the daily exposure dose of trace elements in dusts.

Parameters	Unit	Values
Heavy metal concentration (C)^a^	mg kg^−1^	
Skin-surface area (SA)^a^	cm^2^	5800
Exposure frequency (EF)^a^	day year^−1^	350
Exposure duration (ED)^a^	year	30
Conversion factor (CF)^a^	L cm^−3^	0.07
Body weight (BW)^b^	kg	70
ABS^a^		0.1
Average time (AT) ^a^	Days	For non-carcinogenic risk: ED×365
		For carcinogenic risk: 70 × 365
Particle emission factor (PEF)^a^	kg m^−3^	1.36 × 10^9^
Ingestion rate ingR^a^	mg day^−1^	100
Inhalation rate inhR^a^	day m^−3^	20

References: a = DoEA [43]; b = USEPA [47].

Non-carcinogenic risk can also be characterized by another term hazard index (HI), which is the combined contribution of ingestion, inhalation, and dermal contact doses of a specific heavy metal in a medium described by United States Environmental Protection Agency [47]. The mathematical representation of this parameter:

(5) $$\text{HI}=\left(\frac{\text{Di}}{\text{RfD}}\right)\text{ingestion+}\left(\frac{\text{Di}}{\text{RfD}}\right)\text{imhalation+}\left(\frac{\text{Di}}{\text{RfD}}\right)\text{dermal}$$

Where *D_i_* and *RfD* are daily doses and reference for element *i* and corresponding reference dose of that element respectively. HI is used to estimate the risk of elements due to ingestion, inhalation, and dermal contact. HI ≤ 1 indicated no adverse health effects and HI > 1 indicated likely adverse health effects [47].

### 2.7 Carcinogenic risk estimation

The carcinogenic risks can be expressed by the incremental probability that an individual will develop cancer over a lifetime of the exposure to a specific contaminant or potential carcinogen [45]. As with non-cancer risks characterization, carcinogenic risk characterization also helps to quantify risks using contaminant intakes and toxicity values [47]. For carcinogens, the dose was multiplied by the corresponding cancer slope factor to produce an estimated cancer risk. Risk management decisions were most frequently made when the cancer risk ranges were 10^−6^ to 10^-4^ [47].

The following equation which is used for calculating the cancer risk analysis:

(6) Risk = LADD_i_ × CSF_i_

Where Risk is a unit less probability of an individual developing cancer over a lifetime. LADD_i_ (mg kg^−1^ day^-1^) and CSF_i_ (mg kg^−1^ day^-1^)^-1^ are the average daily exposure over a life time and the cancer slope factor for element i, respectively.

### 2.8 Statistical analysis

A bivariate statistical analysis has been performed to assess the influences of smoking status on the four metal (Pb, Cd, Ni and Zn) concentrations. Moreover, *t*-test has also been done to check the significance of this bivariate analysis. The results of the statistical analysis have been discussed in Section 3.3.

## 3 Results and discussion

### 3.1 Overview of the heavy metal concentrations

The concentration of heavy metals was assessed in all the blood samples for Pb, Cd, Zn, Ni, and Cr using atomic absorption spectrophotometer (AAS). For heavy metal contamination at the exposed site, workers were categorized into two different sets, smoking habits and age groups. The concentration level ranges and total averages of Pb, Cd, Ni, and Zn were 14.50–48.00 (32.78), 0.35–2.05 (1.08), 0.40–4.90 (1.42), and 6.55–14.75 (9.08) μgL^−1^, respectively (Appendix - Table 1). There were few samples having a concentration of the bellow detection limits for Pb, Cd, Ni, and Zn. But Cr concentration in all samples was bellow detection limit. The trend of the toxicity of the heavy metals in human blood as follows: Pb > Zn > Ni > Cd. A similar study was performed by Sani & Abdullahi [25] for heavy metals in the body fluids of the workers in metal industries. Cd had the lowest concentration, whereas Pb had the highest concentration in the blood fluids of the workers, and followed the sequence of Pb > Ni > Mn > Cr > Cd [25].

The reason for the high concentration of Pb in the workers could be attributed to the plastic materials such as PVC based plastic work with lead-containing stabilizers, including dibasic lead phthalate, lead chlorosilicates and basic hard lead carbonates, all of which can produce dust when agitated [32]. Laboratory investigation of urine samples by Atomic absorption spectrophotometry in blood samples, there were higher concentrations of Manganese (Mn), Lead (Pb), Chromium (Cr), and Nickel (Ni). Metal workers of urban Kano are at risk because of the concentration of Mn and Pb in particular. There is a need to monitor occupational activities that are responsible for pollution and with serious health risks.

### 3.2 Heavy metals speciation between smokers and non-smokers

The ranges and mean concentration of heavy metals (Pb, Cd, Ni, and Zn) in blood specimen of the smoker workers were 19.00–48.00 (33.75), 0.40–1.75 (1.11), 0.40–4.90 (1.58), and 7.30–14.75 (9.11) μgL^−1^, respectively (Table 3). For non-smokers, the ranges and average concentration of heavy metals were 14.50–46.00 (31.28), 0.45–2.05 (1.04), 0.45–3.15 (1.21), and 6.55–13.50 (9.05) μgL^−1^ for Pb, Cd, Ni, and Zn, respectively (Table 3). The concentration of the investigated result shows that the levels of the metals in the non-smoker human blood fluctuated within a relatively narrow range for the particular element.

**Table 3:** Summary of the Heavy metal concentrations in blood samples between smokers and non-smokers workers in plastic industry, Dhaka, Bangladesh. All units are in μgL^−1^.

Metals	Lead (Pb)		Cadmium (Cd)		Nickel (Ni)		Zinc (Zn)	
	Smoker	Non-smoker	Smoker	Non-smoker	Smoker	Non-smoker	Smoker	Non-smoker
Average	33.75	31.28	1.11	1.04	1.58	1.21	9.11	9.05
Minimum	19.00	14.50	0.40	0.45	0.40	0.45	7.30	6.55
Maximum	48.00	46.00	1.75	2.05	4.90	3.15	14.75	13.50
STDEV	9.19	10.26	0.59	0.44	1.17	0.77	2.08	1.88
p-value	0.553		0.732		0.379		0.942	

From the results of the bivariate analysis and t-tests (p-values has given in Table 3), it is clear that smoking status does not make any influence on the concentrations of these four metals as all the p-values are very high (p > 0.05). As the difference between these two groups (smoker and non-smoker) is negligible and the p-value is 0.553 implies there exists no significant association between smoking status and concentration of Pb, Cd, Ni, and Zn. Some fluctuations of heavy metal concentration in each individual workers come from the variety of related parameters of the human subjects, namely environment, food, age, culture, and habits [21,33].

### 3.3 Heavy metals speciation among different age group of workers

**Fig. 2:**
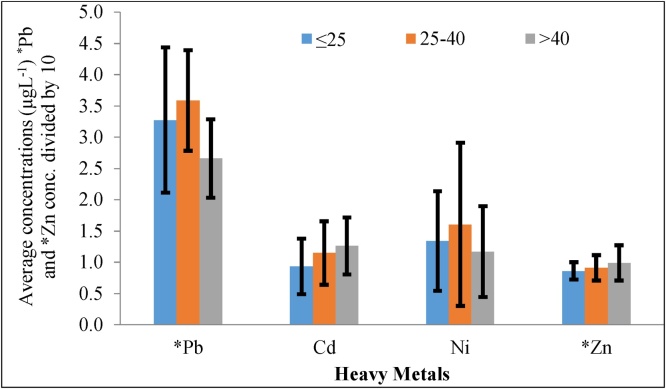
The average concentrations ((μgL^−1^) of heavy metals in blood samples of three different age groups (I = ≤25, II = 26–40, and III = >40 years) workers in the plastic industry, Dhaka, Bangladesh. *=Pb and *Zn concentrations were divided by 10 for better visualization.

**Fig. 3:**
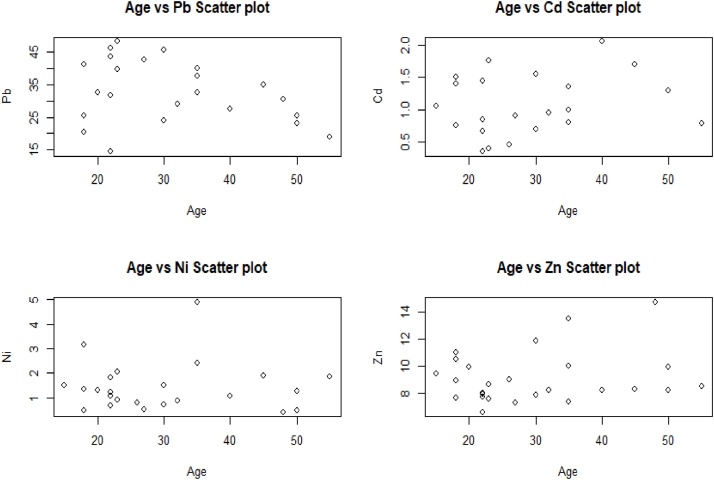
Scatter plot of heavy metals concentrations with respect to age of the respondents in the plastic industry, Dhaka, Bangladesh.

Fig. 2 shows an overall outcome of this study according to age groups for the selected metals measured in blood samples. The ages of the workers were classified into three sets of groups ≤ 25 (I), 26–40 (II), and >40 (III) years. The average concentration with standard deviation and ranges of the selected heavy metals for a different set of groups are (26.6 ± 6.28–35.90 ± 8.60), (0.93 ± 0.44–1.26 ± 0.46), (1.17 ± 0.73–1.61 ± 1.31), and (8.62 ± 1.38–9.91 ± 2.80) for Pb, Cd, Ni, and Zn, respectively. The concentration of Pb, Cd, Ni, and Zn varied as the age group with different tendency and the concentration decreased in the order of II (26–40) >I (≤25) >III (= >40 y) for Pb and Ni and III (= >40 y) >II (26–40) > I (≤25) for Cd and Zn, respectively (Fig. 2). Fig. 3 shows the scatter plot of respondent’s age with metals concentration and Table 4 represents the correlations between age and metal concentrations.

**Table 4:** Correlation analysis of metals concentrations with respect to the Age of the respondents in Plastic Industry, Dhaka-Bangladesh.

Concentrations	Correlation Co-efficient	p-value
Pb	−0.318	0.139
Cd	0.208	0.353
Ni	−0.006	0.976
Zn	0.203	0.320

From the Fig. 3, no significant pattern has been visible on any of these four sub-plots. As a consequence, Table 4 represents no significant correlation between age and concentration as p-values are much higher. For example, the correlation coefficient of age and Zn concentration is 0.203 with a p-value 0.320 (>0.05). This shows there is a weak positive correlation between age and Zn concentration. A literature review of the heavy metal concentrations of the various industries in different countries of the World has given for the comparison in Table 5.

**Table 5:** Concentrations of heavy metals in Blood samples of workers in different occupational settings in the World (Unit: μgL^−1^).

Location	Industry Category	Pb	Cd	Ni	Zn	Methods
Dhaka, Bangladesh^a^	Plastic industry	32.78	1.08	1.42	9.08	AAS
Lahor, Pakistan^b^	Steel Mills	263.0	5.16		8.54	AAS
Kirkuk, Iraq^c^	Gas company	25.4	1.09			AAS
Sialkot, Pakistan^d^	Leather tanning, products & manufacturing	119.0	9.0	4.18	22.3	FAAS
Granada, Spain^e^	Iron and steel	43.39	0.49	0.96		ETAAS
Sialkot, Pakistan^f^	Surgical industry	25.18	5.61	4.66	13.5	FAAS

a = this study; b = Afridi et al. [48]; c= Husien et al. [49]; d=Junaid et al. [16]; e=Kurt-Karakus [51]; f=Junaid et al. [50].

### 3.4 Concentrations of heavy metals in dust samples

The concentration of heavy metals in dust samples collected from three different industrial sections of the plastic industry is summarized in Table 6. A relatively higher level of toxic heavy metal concentrations of Pb (47.24 mg kg^−1^) in the pellet manufacturing section and Cd (4.20 mg kg^−1^) in the plastic waste recycling section were found, which have been reported in many similar studies [9,34]. When the recycling activities of plastic wastes, especially milling, washing, and extrusion processes are not controlled properly, heavy metals could be released and becomes one of the major contributors of heavy metals to the environment [9]. Other heavy metals Zn and Ni were found higher in concentration (112.54 and 21.56 mg kg^−1^) in the pellet manufacturing section of the industry.

**Table 6:** Heavy metals concentrations (mgkg^−1^) of dust samples in three different sections of the plastic industry, Dhaka-Bangladesh. P1 = Plastic waste recycling section, P2 = Pellet manufacturing, P3 = Plastic goods manufacturing section.

Metals	Industrial Sub-sections			Mean	STDEV
	P1	P2	P3		
Pb	45.18	47.24	28.40	40.27	10.33
Cd	4.20	2.63	2.95	3.26	0.83
Ni	14.35	21.56	18.35	18.08	3.61
Zn	96.50	112.54	101.90	103.64	8.16

### 3.5 Non-carcinogenic risk analysis

To specify the daily intake frequencies of the indoor industrial dust and related health risks, the acceptable daily intake (ADI), hazard quotient (HQ), and hazard index (HI) of investigated heavy metals through the intake of indoor industrial dust by the exposed workers have been calculated. The acceptable daily intake (ADI), hazard quotient (HQ), and health index (HI) for non-carcinogenic risk of heavy metals through ingestion, inhalation, and dermal contact in different industrial subsections have given in Table 7. Acceptable daily intake (ADI) for the ingestion pathway is higher than those obtained for inhalation and dermal exposures for all recruited workers in this study, followed by the same sequence for hazard quotient (HQ). So, workers in this plastic industry were more at risk of non-carcinogenic effects when exposed to ingestion than the other two modes of exposure pathways of heavy metal contamination. For heavy metals in dust samples the estimated average daily intake dose range from (3.09 × 10^−9^ -5.52 × 10^-5^), (2.48 × 10^-10^ – 4.46 × 10^-6^), (1.37 × 10^−9^ – 2.47 × 10^-5^), and (7.88 × 10^-6^ – 1.42 × 10^-4^) for Pb, Cd, Ni and Zn, respectively for all three pathways (Table 7). Nduka et al. [35] studied on the health risk assessment of heavy metals from car workshops, and found potential cancer risk through inhalation for Cd and Cr for both children and adults. The values were above the US EPA limit values of 1 × 10^-6^ to 1 × 10^-4^. These high-level exposure to heavy metals may compromise the body’s immune system of the workers in the car workshop [35]. The chronic daily intake with carcinogenic and non-carcinogenic health risk assessment of heavy metals (Pb, As, Cd, Hg, and Ni) from painkiller drugs were also studied in Nigeria [36]. No significant difference was observed between carcinogenic and non-carcinogenic risk except minor variation. The non-carcinogenic risk of chronic daily intake was in the range of 10^-6^ and 10^−9^ [36].

**Table 7:** Health Risk Assessment of heavy metals exposure in the plastic industry, Dhaka-Bangladesh. R*f*D = Reference Dose (mgkg^−1^-day), CSF = Chronic Reference Dose, ADI = Acceptable Daily Intake, HQ = Hazard Quotient, HI = Hazard Index, CR = Carcinogenic Risk.

Element	RfD_ing_	RfD_inh_	RfD_derm_	CSF_inh_	ADI		HQ	HI	CR
**^a^Pb_non-cancer_**	3.50E-03	3.50E-03	5.25E-04		Ingestion Inhalation Dermal	5.52E-05 3.06E-09 2.24E-06	1.58E-02 8.74E-07 4.27E-03	2.00E-02	
**^a^Pb_cancer_**				4.20E-02					1.46E-10
**^a^Cd_non-cancer_**	1.00E-03	6.30E+00	1.00E-03		Ingestion Inhalation Dermal	4.46E-06 2.48E-10 1.81E-07	4.46E-03 3.94E-11 1.81E-04	4.64E-04	
**^c^Cd_cancer_**				6.30E+00					1.77E-09
**^a^Ni_non-cancer_**	2.00E-02	2.06E-02	5.40E-03		Ingestion Inhalation Dermal	2.47E-05 1.37E-09 1.01E-06	1.24E-03 6.65E-07 1.87E-04	1.42E-03	
**^b^Ni_cancer_**				8.40E-01					1.31E-09
**^a,d^Zn**	3.00E-01	3.00E-01	7.50E-02		Ingestion Inhalation Dermal	1.42E-04 7.88E-09 5.76E-06	4.73E-04 2.63E-08 7.68E-05	5.49E-04	

References: a = DoEA [43]; b = [31]; c = USEPA [45]; d = USEPA, 2015.

However, the HQ values <1 are considered as harmless with no minimal risks to the health of the exposed workers with that exposure level to certain heavy metal, while the values HQ > 1 portray the possibility of serious health risks, which can lead to the induction of diseases. Moreover, according to the health risk classification of the USEPA [[47]], when the HI value of heavy metal exposure >1, the associated risk becomes higher. In this study, the mean HI values were <1 for all three different subsections of the investigated plastic industry indicates different exposure pathways to the individual metals bear no significant risk of non-carcinogenic effects (Table 7).

### 3.6 Carcinogenic risk analysis

The US Environmental Protection Agency (USEPA) considers acceptable or tolerable threshold limit value for cancer risk is ranged from 1.0 × 10^−6^ to 1.0 × 10^-4^ [[47]]. The risk estimation from a single sources of individual chemical or mixture of chemicals has significant limitations with current HQ and HI [37]. Each metal displays different modes of toxicity and the cumulative effect of a mixture of metals could be undervalued. Therefore, the risk analysis should be improved with sensitive analytical techniques as well as the modification of risk assessment approaches [37]. However, the carcinogenic risk was calculated based on Cd and Pb concentration with the EPA method and found to be the highest contribution (2.02 × 10^-7^) to the cancer risk (Table 7). The ingestion route seems to be the major contributor to cancer risk to the exposed industrial workers. This study shows the potential health risks of heavy metal exposures to the industry workers. Therefore, the study population groups need long term clinical follow up to assess their health status, laboratory works to follow their liver and renal function as most of the excretion happen from the body through the liver and kidney. Also, cardiovascular neurological, and hematological work up recommended for establishing the long term effects and co-morbidities of the industry workers.

## 4 Conclusion

Plastic industries have been growing rapidly in all over the world and exert a serious problem to the environment. Occupational exposure to the elevated levels of heavy metals associated with health risks in the exposed workers of the plastic industry had not been testified extensively previously. Therefore, the focus of this study was to determine the levels of heavy metals levels in the blood of exposed workers and their potential health consequences. The ascetically elevated levels of heavy metals concentration in biological matrices of the exposed workers were detected in this plastic industry. Dust from different industrial activity zones comprised higher concentrations of Zn, Pb and Ni. Parameters (e.g., ADI, HQs, and HI) indicated that exposed workers had relatively less critical health consequences of the heavy metals pollution. Nevertheless, when these heavy metal levels will be elevated and came into contact with environmental commodities as untreated waste, they will cause devastating and drastic effects. Therefore, proper improved working conditions for the exposed labor and treatment of industrial wastes to secure environmental degradation in the industrial vicinities are strongly recommended.

## Author’s contribution

Md. Shakil Ahmed: Sampling, Chemical analysis and drafting manuscript

Mahbuba Yesmin: Drafting and reviewing manuscript

Farah Jeba: Drafting and reviewing manuscript

Md Sirajul Hoque: Supervision, chemical analysis, research concept

Ahsan Rahman Jamee: Data analysis and manuscript reviewing

Abdus Salam: Supervision, data analysis and manuscript reviewing

## Declaration of Competing Interest

The authors report no declarations of interest.

## Figures and Tables

**Table A1:** Concentrations of heavy metals (Pb, Cd, Ni, Zn)) of individual blood samples in the plastic industry, Dhaka, Bangladesh. Chromium (Cr) concentrations were bellow detection limit in each sample. All units are in μgL^−1^.

Sample No.	Lead (Pb)	Cadmium (Cd)	Nickel (Ni)	Zinc (Zn)
1	42.50	0.90	0.50	7.25
2	35.00	1.70	1.90	8.25
3	46.00	0.85	0.65	6.55
4	24.00	0.70	0.70	7.80
5	20.50	1.40	BDL	7.60
6	BDL	0.75	3.15	11.00
7	32.50	1.00	BDL	13.50
8	25.50	BDL	0.45	9.90
9	BDL	0.45	0.80	9.00
10	45.50	1.55	1.50	11.85
11	40.00	1.35	4.90	7.30
12	37.50	0.80	2.40	9.95
13	23.00	1.30	1.25	8.15
14	19.00	0.79	1.85	8.50
15	30.50	BDL	0.40	14.75
16	27.50	2.05	1.05	8.15
17	39.50	0.40	0.90	8.65
18	48.00	1.75	2.05	7.50
19	BDL	1.05	1.50	9.40
20	31.50	0.35	1.20	7.65
21	41.00	BDL	1.35	10.50
22	29.00	0.95	0.85	8.20
23	25.50	1.50	0.45	8.90
24	14.50	0.66	1.80	8.00
25	43.50	1.45	1.05	7.90
26	32.50	BDL	1.31	9.90
**Ave**	**32.78**	**1.08**	**1.42**	**9.08**
**STD**	**9.47**	**0.47**	**1.01**	**1.95**
**Min**	**14.50**	**0.35**	**0.40**	**6.55**
**Max**	**48.00**	**2.05**	**4.90**	**14.75**

## Appendix A

See Table A1
